# Longitudinal changes in functional connectivity in speech motor networks in apraxia of speech after stroke

**DOI:** 10.3389/fneur.2022.1013652

**Published:** 2022-11-30

**Authors:** Helena Hybbinette, Per Östberg, Ellika Schalling, Catharina Deboussard, Jeanette Plantin, Jörgen Borg, Påvel G. Lindberg

**Affiliations:** ^1^Division of Speech and Language Pathology, Department of Clinical Science, Intervention and Technology, Karolinska Institute, Stockholm, Sweden; ^2^Division of Rehabilitation Medicine, Department of Clinical Sciences, Danderyd Hospital, Karolinska Institute, Stockholm, Sweden; ^3^Department of Rehabilitation Medicine, Danderyd University Hospital, Stockholm, Sweden; ^4^Medical Unit Speech and Language Pathology, Karolinska University Hospital, Stockholm, Sweden; ^5^Department of Public Health and Caring Sciences, Speech-Language Pathology, Uppsala University, Uppsala, Sweden; ^6^Institut de Psychiatrie et Neurosciences Paris, INSERM U1266, Université de Paris, Paris, France

**Keywords:** apraxia of speech, functional connectivity, resting-state fMRI, stroke, recovery

## Abstract

**Objective:**

The cerebral substrates of apraxia of speech (AOS) recovery remain unclear. Resting state fMRI post stroke can inform on altered functional connectivity (FC) within cortical language networks. Some initial studies report reduced FC between bilateral premotor cortices in patients with AOS, with lowest FC in patients with the most severe AOS. However, longitudinal FC studies in stroke are lacking. The aims of the present longitudinal study in early post stroke patients with AOS were (i) to compare connectivity strength in AOS patients to that in left hemisphere (LH) lesioned stroke patients without a speech-language impairment, (ii) to investigate the relation between FC and severity of AOS, aphasia and non-verbal oral apraxia (NVOA) and (iii) to investigate longitudinal changes in FC, from the subacute phase to the chronic phase to identify predictors of AOS recovery.

**Methods:**

Functional connectivity measures and comprehensive speech-language assessments were obtained at 4 weeks and 6 months after stroke in nine patients with AOS after a LH stroke and in six LH lesioned stroke patients without speech-language impairment. Functional connectivity was investigated in a network for speech production: inferior frontal gyrus (IFG), anterior insula (aINS), and ventral premotor cortex (vPMC), all bilaterally to investigate signs of adaptive or maladaptive changes in both hemispheres.

**Results:**

Interhemispheric vPMC connectivity was significantly reduced in patients with AOS compared to LH lesioned patients without speech-language impairment. At 6 months, the AOS severity was associated with interhemispheric aINS and vPMC connectivity. Longitudinal changes in FC were found in individuals, whereas no significant longitudinal change in FC was found at the group level. Degree of longitudinal AOS recovery was strongly associated with interhemispheric IFG connectivity strength at 4 weeks.

**Conclusion:**

Early interhemispheric IFG connectivity may be a strong predictor of AOS recovery. The results support the importance of interhemispheric vPMC connection in speech motor planning and severity of AOS and suggest that also bilateral aINS connectivity may have an impact on AOS severity. These findings need to be validated in larger cohorts.

## Introduction

Apraxia of speech (AOS) is traditionally defined as a disorder of speech motor planning and programming. The disturbance is regarded to be located at the interface between the linguistic formulation phase and speech motor execution, reflecting “inefficiencies in the translation of well-formed and -filled phonological frames into previously learned kinematic information” (1). The core symptoms of AOS include articulatory errors that are perceived as sound and/or syllable distortions, slow speech rate with extended segment and inter-segment durations and prosodic deficits (2, 3). A stroke in the language-dominant (typically left) hemisphere within the territory of the middle cerebral artery (MCA) is considered the most frequent cause of AOS. Less frequent etiologies are traumatic brain injury, tumors, and neurosurgery (3). Apraxia of speech can also be caused by neurodegenerative disease, often as part of the non-fluent/agrammatic variant of primary progressive aphasia (PPA) (4). When progressive AOS is the only or the primary neurological deficit, it is referred to as primary progressive apraxia of speech (PPAOS) (5, 6). AOS after stroke rarely occurs in isolation; rather, it is most often accompanied by aphasia and/or dysarthria (3, 7, 8). Many patients with AOS also have non-verbal oral apraxia (NVOA) (9), especially severe AOS tends to co-occur with NVOA (3). The frequent association indicates that the mechanisms for oromotor and speech motor control to some degree depend upon shared substrates (10).

There is a consensus today that the pathogenesis of AOS is associated with disturbances to a network of brain regions. Several different regions have been proposed to be involved, but the exact constituents and the role and relationship between the sites and pathways in this network remain unclear (11–13). Early post-mortem studies identified an area in the posterior inferior frontal gyrus of the dominant hemisphere (“Broca's area”) as a region associated with speech articulation difficulties (14), a localization that since then has gained rich support in AOS research, for example by Richardson et al. (15), Trupe et al. (16) and Wertz et al. (17). Dronkers (18) instead concluded that the left insula is the most crucial area for motor speech planning/programming and its lesion contributes to AOS. This was later questioned by other researchers. Hillis et al. (11) argued that the association between insular damage and AOS might primarily relate to the vulnerability of the insula to large MCA strokes. That there is an association between left anterior insular lesions and AOS was not disputed, but its precise role in speech production was viewed as unclear. Eickhoff et al. (19) suggested that the insula could serve as a relay between cognitive aspects of language and the preparation of speech motor movements. Based on a meta-analysis of published functional neuroimaging studies and own fMRI data, they proposed that the phonetic concept of an intended speech act, presumably deriving from Broca's region, would be received in the insula where the information is encoded into articulatory motor patterns. The insula forwards the plan to the cerebellum and the basal ganglia, which both project to the premotor cortex. The premotor cortex transforms the intended actions into specific patterns for muscle activation, that then are forwarded to primary motor cortex and on to lower motor neurons and the final execution phase (19). It has been shown that in patients with stroke-induced acute AOS, the premotor cortex was the region predominantly associated with AOS symptoms (8). The importance of the premotor cortex in speech production has also been highlighted in neurocomputational models of speech production, such as the Directions Into Velocities of Articulators (DIVA) model (20) and its expanded version, the Gradient Order DIVA (GODIVA) model (21, 22). According to these models, a lesion in the left premotor cortex would impair the conversion of well-formed phonological messages into previously learned speech motor movements and thereby account for the speech programming impairment associated with AOS. Also in patients with PPAOS, abnormalities in premotor and supplementary motor regions are considered as highly plausible candidates responsible for AOS symptoms (23).

The role of the right (or non-dominant) hemisphere in speech-language function and its ability to compensate after a left hemisphere (LH) stroke has been long debated. It is well established that areas in the right hemisphere (RH), homologous to LH speech-language areas, are activated during speech-language activities in healthy adults. This activation has been shown to increase depending on task difficulty and degree of cognitive load (24, 25). However, it is still not clear whether additional activation of RH regions is beneficial or maladaptive for speech-language recovery after a stroke or an acquired brain injury. Some studies argue that good language recovery can only be obtained by a reactivation in the LH, while others describe a positive recovery by activation in the RH. The activation pattern may also change during the different stages after the stroke and factors such as lesion size and site are assumed to be involved (26–28). Functional brain reorganization with an increased activity in homologous areas in the RH has also been observed in individuals with PPA and PPAOS (29, 30).

Resting state functional magnetic resonance imaging (rs-fMRI) enables study of functional network connectivity (31). Low frequency (<0.1 Hz) BOLD signals acquired during rest show correlated activation within anatomically separated brain regions believed to reflect networks that typically are engaged in shared functions (32–34). While conventional fMRI compares changes in the BOLD signal during a task period and a baseline state, rs-fMRI is obtained in the absence of a stimulus or a task with the patient simply “resting” in the scanner. The technique is therefore considered particularly suitable for individuals who may have difficulty to perform a certain task, for example stroke patients with a speech-language impairment (27). There are several methods to analyze functional connectivity (FC) data. In a seed-based analysis approach, FC is evaluated by computing cross-correlations between different regions of interest (ROI). This method requires a priori selection of ROIs and is often based on a hypothesis or prior results (35).

To the best of our knowledge, only one published study has applied rs-fMRI to investigate FC in patients with AOS after stroke. New et al. (36) studied thirty-two post stroke aphasia patients, fifteen of which had concomitant AOS. The time post stroke onset among the participants with AOS varied between 1 to 156 months, with the majority being in a chronic phase. Functional connectivity was examined based on the DIVA model, in a network of regions proposed as key areas in speech production: the inferior frontal gyrus (IFG), anterior insula (aINS), and ventral premotor cortex (vPMC) (37). To study the relation between the affected and unaffected hemisphere, both hemispheres were included in the analyses. When comparing the whole patient group with eighteen healthy controls, reduced FC between each interhemispheric homotopic seed region was found in the patient group. Comparisons between patients with and without AOS showed that patients with AOS had reduced FC between bilateral vPMC and between left vPMC and right aINS. The reduced FC between interhemispheric homotopic vPMC regions was related to AOS severity, while a negative FC between the left vPMC and right aINS related to NVOA severity.

Beside the study of stroke patients by New and colleagues, Botha et al. (38) studied FC in twenty-two patients with PPAOS. For the connectivity analyses, a hybrid method was applied by choosing a set of hypothesis-driven, predefined Intrinsic Connectivity Networks (ICN) from the Mayo Clinic Study of Aging (MCSA) functional connectivity atlas (39). The main reported finding was a reduced connectivity between the right supplementary motor area (SMA) and left posterior temporal lobe to the rest of the speech and language ICN network, with the degree of reduced connectivity negatively correlated with an articulatory error score (38).

In addition to the limited number on FC and its relation to AOS, few studies have investigated AOS in early stroke patients and how the symptoms evolve over time. Most studies on AOS are focused on patients in a chronic stage, often with milder impairments than those that may occur at an early stage, and factors that may predict recovery are largely unknown (7, 40). The overall aim of this study was thus to address the recognized knowledge gap regarding cortical connectivity within a speech production network related to AOS. Based on previous findings, the specific aims were:

1) To compare FC strength in patients with AOS after a LH stroke to that in LH lesioned stroke patients without a speech-language impairment, at a subacute phase at 4 weeks and at a chronic phase at 6 months.
2) To investigate the relation between FC and degree of severity in AOS, aphasia and NVOA.
3) To investigate longitudinal changes in FC in post stroke patients with AOS with concomitant aphasia and NVOA, from the subacute to the chronic phase, to identify predictors of AOS recovery.

## Materials and methods

### Participants

The participants were recruited from a group of post stroke patients participating in a longitudinal study of hand motor function, the ProHand Study (ClinicalTrials.gov Identifier: NCT02878304). The study was approved by the Regional Ethical Review Board in Stockholm and informed consent was obtained from all participants. To enable the inclusion of patients with impaired language abilities, both oral and written information was adapted and presented in an aphasia-friendly manner. Inclusion criteria were: 1) Patients aged ≥18 years admitted to inpatient care after first ever-stroke, 2) Swedish as first language, 3) Time of enrollment: between 2 and 6 weeks after stroke onset, 4) Clinical evidence of hand motor deficits based on neurological examination and medical records, 5) Awake, alert and capable of participating in assessment procedures. Exclusion criteria were: 1) Inability to understand and comply with instructions (presented in an adapted format for patients with aphasia), 2) Cerebellar lesions, 3) Report of claustrophobia or metal objects in the body, 4) Presence of other neurological, psychiatric, or medical conditions that preclude active participation. Brain imaging examination with sequences for resting state fMRI and behavioral assessments were conducted at two time points: the first (A1) on average at 4 weeks after stroke onset (mean 33 days, range 24– 42 days), and the follow-up (A2) at 6 months post stroke onset (range ± 9 days). Nine patients with AOS at A1 with complete data from both assessments were included in the longitudinal study. All in this group also had aphasia and NVOA at A1, all also had mild to severe upper limb motor impairment. Four of the participants also had unilateral upper motor neuron dysarthria, but to such a mild degree that it did not interfere the other speech-language measurements. To enable comparison of FC patterns between LH lesioned patients with AOS to the FC in LH patients without AOS symptoms, a control group including six LH lesioned patients with no signs of AOS, aphasia or NVOA was recruited from the same longitudinal cohort. The AOS and the non-AOS comparison group did not differ regarding age or sex but differed significantly in level of hand motor impairment. For descriptives and clinical characteristics, see section “Characterization of lesion data” and **Table 3**.

Between the two assessments, all participants received team-based rehabilitation. During the stay at the inpatient clinic, which lasted between ten to twelve weeks for all the participants in the AOS group, speech-language treatment was provided according to guidelines by the National Board of Health and Welfare in Sweden with four to five sessions a week of individual SLP training. These sessions were in general 30–45 min long and included interventions targeting both language and speech outcomes on a functional and structural level, based on the individual participant impairment profile. Interventions that addressed communication activities and participation based on personal goals were also included in the sessions. After discharge from the inpatient clinic, all participants received speech-language interventions provided by their regional rehabilitation team. These interventions were mainly homebased, with SLP training two to three times a week. No participant received highly intensive speech-language therapy in any form, such as Intensive Language Action Therapy (ILAT) (41).

### Behavioral assessment

Presence and degree of AOS was investigated using the Apraxia of Speech Rating Scale 2.0 (ASRS 2.0) (42, 43). The ASRS 2.0 includes ratings of thirteen characteristics on a 5-point scale. Maximum total score is 52 and the recommended cut-off value for an AOS diagnosis is ≥8 points (42). The descriptors for each level of rating are: (0) not observed in any task/no more than one occurrence”; (1) “infrequent/noted more than once”; (2) “frequent but not pervasive/noted in 20–50% of all utterances, but not on most tasks or utterances”; (3) “nearly always evident but not marked in severity/noted on many utterances on most tasks but not enough to decrease overall intelligibility” and (4) “nearly always evident and marked in severity/noted on most utterances on most tasks and severe enough to impact intelligibility.” The ASRS is increasingly used in studies of AOS, as for example by Clark et al. (44), Mailend et al. (45) and Staiger et al. (46), and the total score has been found to be a reliable measure of AOS severity after stroke (47). However, some of the items on the ASRS require a certain level of speech production to be ratable in accordance with the formulations of level descriptors. Problems to confidently score patients with signs of severe AOS with the ASRS have therefore been noted (48). To enable the use of the ASRS in this study and ratings that reflected the observed severity levels also for participants with a limited speech production, adapted strategies for ratings in four of the thirteen items on the ASRS were used. These modifications are presented in Table 1. The complete ASRS can be seen in Utianski et al. (49).

**Table 1:** Modified ratings for participants with limited speech production on the ASRS 2.0.

**0**	**1**	**2**	**3**	**4**
**Not observed in any task**	**Infrequent**	**Frequent but not pervasive**	**Nearly always evident but not marked in severity**	**Nearly always evident and marked in severity**
No more than one occurrence	Noted more than once	Noted 20–50% of all utterances, but not on most tasks or utterances	Noted on many utterances on most tasks but not enough to decrease overall intelligibility	Noted on most utterances on most tasks and severe enough to impact intelligibility
**Items on the ASRS 2.0**			**Applied rating strategies**	
1.3 Increased sound distortions or distorted sound substitutions with increased utterance length or increased syllable/word articulatory complexity			For individuals who cannot produce phrases or multisyllabic words, but these symptoms are noticed in monosyllabic words and in isolated speech sounds, a score of 4 is applied.	
2.1 Syllable segmentation within words > 1 syllable (Brief silent interval between syllables and/or inappropriate equalized stress across syllables)			For individuals who cannot produce multisyllabic words but shows apraxic symptoms that are judged be the underlying course of the impairment, a score of 1–4 is applied^*^	
2.2 Syllable segmentation across words in phrases/sentences (Increased inter-word intervals and/or inappropriate equalized stress across words)			For individuals who cannot produce phrases/sentences but shows apraxic symptoms that are judged be the underlying course of the impairment, a score of 1–4 is applied^*^	
3.1 Rate only for SMRs: Deliberate, slowly sequenced, segmented (gaps between sequences), and/or distorted (including distorted substitutions) speech SMRs in comparison to AMRs. *Rate the best effort*Score on severity only: 0 = not noted, SMRs normal; 1 = slow, 2 = mildly segmented and/or distorted; 3 = moderately segmented and/or distorted, 4 = severely segmented and/or distorted			For individuals who have major problems producing both AMR and SMRs and show apraxic symptoms that are judged be the underlying course of the impairment, a score of 4 is applied.	

* Rating value corresponding to degree of observed AOS symptoms being the cause of the impairment, rated in consensus between 2–3 raters/ SLPs.

The AOS symptoms were rated based on the participants' performance during conversation, a picture description task, and by use of a Swedish translation of a motor speech protocol developed at the Mayo Clinic, Supplemental Tasks for Assessing Motor Speech Abilities, described in Duffy et al. (5). These supplemental tasks included measurements of vowel prolongation, repetition of words and sentences of varying length and complexity, as well as production of alternating motion rates and sequential motion rates. Speech production was elicited by the first author or by another speech-language pathologist (SLP) at the same clinic and all sessions were video recorded. The ratings were made by the first author and confirmed by an external SLP with long clinical and research experience of both AOS and aphasia. When discrepancies in scoring occurred, they were discussed and resolved by consensus. Since this study was carried out in a clinical setting, the ratings were also discussed with the clinical SLP, who in some cases also had completed the ASRS for the participant.

Presence, degree, and type of aphasia was assessed by use of the Swedish standardized assessment instrument Neurolinguistic Aphasia Examination (A-ning) (50). A-ning comprises evaluation of seven linguistic modalities: “oral expression abilities,” “repetition,” “auditory comprehension,” “reading comprehension,” “reading aloud,” “dictation” and “informative writing.” The maximum score is 220 points (= no language impairment) and the cut-off for an aphasia diagnosis is <208. Visual confrontation naming ability was assessed by use of the Boston Naming Test (BNT) (51). The maximum result is 60, and scoring was done according to Swedish target words by Tallberg (52). To assess the presence and degree of NVOA, a screening protocol created at the Mayo Clinic was applied. Total maximum score on this screening test is 32 and the recommended cut-off for an NVOA diagnosis is <29 (6, 53).

### Magnetic resonance imaging

Brain imaging at both assessment occasions was performed with an Ingenia 3.0T MR system (www.usa.philips.com) with an 8 HR head coil. High-resolution T1-weighted anatomical images were acquired using TFE 3D (3-dimensional gradient echo-based sequence): field of view, 250 × 250 × 181 mm; matrix, 228 × 227; slice thickness, 1.2 mm; slice spacing, 0.6 mm; and number of slices, 301 (echo time [TE] = 3.456 ms; repetition time [TR] = 7.464 ms). T2 fluid-attenuated inversion recovery (FLAIR) images were also acquired. Resting-state fMRI consisted of a gradient echo-planar sequence (echo time [TE] = 35 ms, flip angle = 90°, voxel size of 1.8 × 1.8 × 4 mm, repetition time [TR] = 3000 ms) sensitive to BOLD contrast. The resting state fMRI sequence lasted 6 min. Patients were instructed to keep eyes closed, to think about nothing in particular, and to not fall asleep. Anatomical T1-images were normalized to Montreal Neurological Institute template using SPM12 (www.fil.ion.ucl.ac.uk/spm/software/spm12/) Clinical toolbox unified segment-normalize procedure (non-linear enantiomorphic normalization, 3-tissue, “old segment,” optimizing the normalization of clinical data with focal brain lesions by exploiting information from homologous regions of the non-lesioned hemisphere). fMRI images were realigned and coregistered to T1 and then normalized using cost function masking to avoid distortion of lesion by normalization procedure (54). The images were inspected visually to ensure adequate normalization. Lesion maps were manually drawn on all axial slices of native space T1 weighted anatomical images using MRIcron (https://people.cas.sc.edu/rorden/mricron/index.html) by a researcher (J.P.) and verified by an experienced neurologist who was blinded to all clinical data. Lesion location was verified on FLAIR images, and lesion maps were binarized. Normalization parameters for T1 images were applied to lesion maps using the SPM12 tool *Old Normalize*.

### ROI selection

The selection of 10 mm diameter ROIs was based on current knowledge on speech motor networks and included regions hypothesized to have an important role in AOS. These ROIs were also applied in the study by New et al. (36): inferior frontal gyrus (IFG), anterior insula (aINS) and ventral premotor cortex (vPMC). Intra- and interhemispheric connectivity between ROIs was studied between similar (homotopic) and different (heterotopic) regions. Montreal Neurological Institute (MNI) coordinates for the selected regions according to Eickhoff et al. (19) are presented in Table 2.

**Table 2:** Coordinates for the selected regions of interest.

**Regions of interest (ROI)**	**MNI coordinates (*x, y, z*) (left–right)**
Inferior frontal gyrus (IFG)	$\begin{array}{ccc} -50\; & 10\; & 5\\ 50\; & 10\; & 5 \end{array}$
Anterior insula (aINS)	$\begin{array}{ccc} -32\; & 15\; & 2\\ 32\; & 15\; & 2 \end{array}$
Ventral premotor cortex (vPMC)	$\begin{array}{ccc} -58\; & 1\; & 23\\ 58\; & 1\; & 23 \end{array}$

### Functional connectivity analyses

BOLD time series from the selected ROIs were extracted as the first principal component of all voxel time series within a spherical (radius 5 mm) volume of interest centered on the individual peak coordinates for each maximum. Image pre-processing included (i) head movement and correction, (ii) co-registration of resting state fMRI (EPI) images to T1-weigted anatomical images, (iii) segmentation (gray matter/white matter/CSF), (iv) normalization using the SPM12 Clinical Toolbox, and (v) smoothing (8 mm). Seed-based FC was calculated using the Connectivity toolbox (55). It incorporates the CompCorr strategy for reduction of noise from physiological and other sources, that takes into account the non-homogeneous distribution of noise signals in the brain. For example, voxels close to white matter or large blood vessels show greater BOLD signal noise. Principal components (PCA) were derived from these noise regions and later included as nuisance parameters within the general linear model. EPI images were inspected visually to identify signal drop-out (due to e.g., the presence of meta-hemoglobin and hemosiderin, i.e., breakdown products from haemorrhagic stroke). Realignment parameters were modeled as regressors of no interest. Estimation of head motion parameters and the presence of image outliers (Artifact Detection toolbox: https://www.nitrc.org/projects/artifact_detect) were included as regressors since it has been shown that this strategy improves motion artifact correction when studying FC (56). Activation threshold of z-normalized global brain signal was set to 3 SD and threshold for rotational and translational head motion was set to 2 mm. This resulted in mean (±SD) = 10 (±11) excluded volumes (out of 160 = 6%). White matter and CSF masks were used for partial volume correction. The principal components of signal from white matter and CSF masks were regressed out during the analysis. A temporal band pass filter (0.01–0.08 Hz) was applied covering approximately the range between 10 and 100 sec which is standard for resting-state connectivity analyses (57). The Conn toolbox computed the average BOLD time series across all the voxels within each ROI. The beta value reflecting interhemispheric and intrahemispheric FC between each pair of ROIs was extracted for each participant. The beta values were then transformed into Fishers- Z values, where each score represents the functional connectivity strength for each connection in each participant.

### Statistical methods

Normal distribution was tested by use of Shapiro-Wilk test and visual inspection. The variables were not substantially skewed, but owing to the limited sample size, non-parametric methods were applied. Between-group differences were tested using the Mann-Whitney U test and Pearson's chi-squared test, whereas within-group differences were assessed with the related-samples Wilcoxon signed-rank test. The degree of recovery was defined as the percentage that a participant improved over time, expressed as a percentage of the maximal possible change: A2-A1/ Max score–A1. Spearman's correlation coefficients were calculated to test the strength of association between FC strength and behavioral measures at four weeks (A1) and 6 months (A2) and recovery (dynamic change over time) in the different speech-language domains. A *p*-value below 0.05 was considered statistically significant. False discovery rate (FDR) (58) was applied to control for multiple comparisons in all univariate correlation analyses with a *q*-value set at <0.003. Statistical analysis was performed by use of the IBM SPSS Statistics for Windows, Version 27.

## Results

### Characterization of lesion data

**Figure 1:**
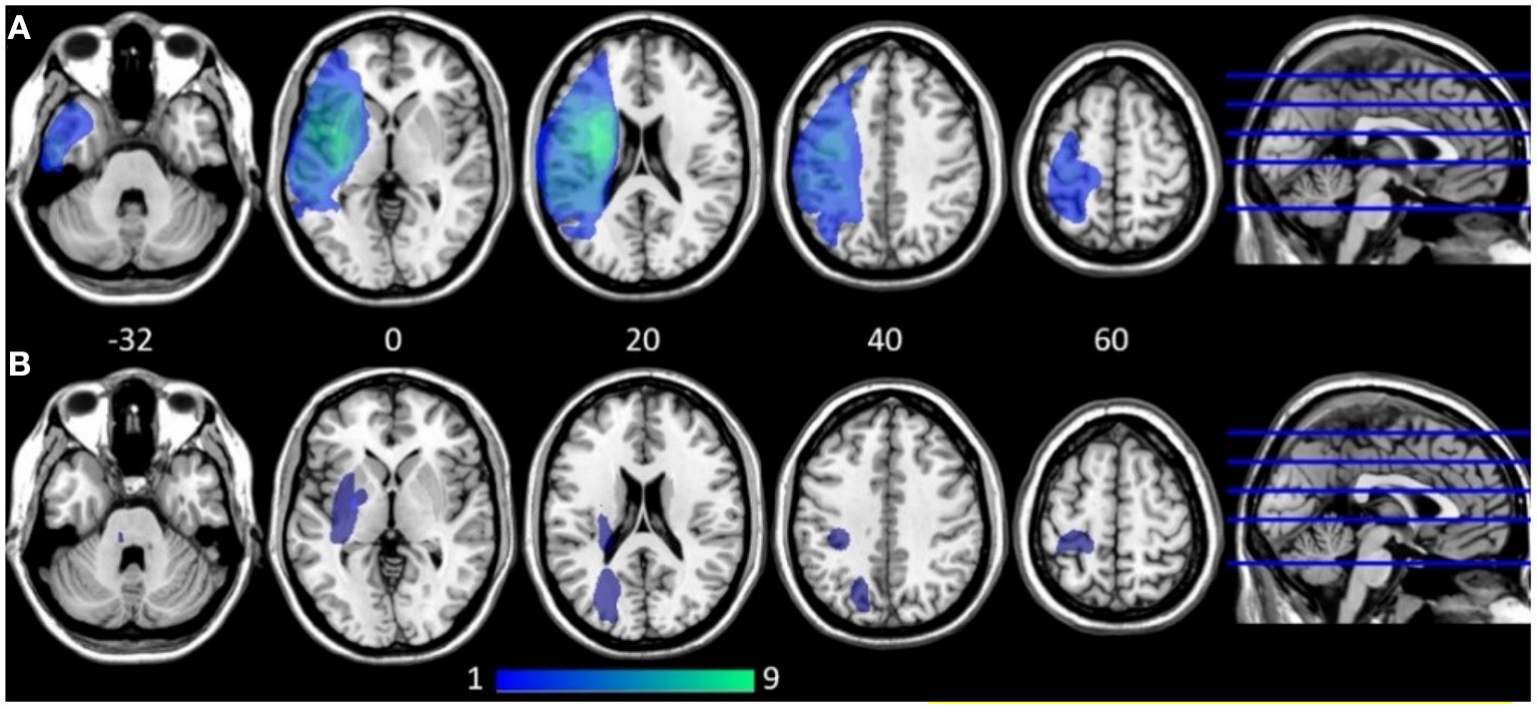
Lesion overlap map at ≈ 4 weeks post stroke onset shown on five axial slices (z coordinates provided). Dark blue indicates low lesion overlap, light green indicates high overlap. **(A)** Participants with apraxia of speech, aphasia, and non-verbal oral apraxia (*n* = 9). **(B)** Participants with no speech-language impairment (*n* = 6). All participants with apraxia of speech, aphasia, and non-verbal oral apraxia had cortical lesions in the middle cerebral artery territory with extensions into deep subcortical white matter where the largest lesion overlap was found. SMA was not lesioned in any of the participants in the AOS group. The participants without speech-language impairment had in general smaller lesions spread in mainly subcortical areas.

All participants with AOS, aphasia, and NVOA (hereafter called the AOS group) had a left MCA stroke with cortical lesions extending into subcortical white matter. The majority were caused by ischemic strokes; one participant had a hemorrhagic stroke. In the group without a speech-language impairment (No SLI group), the lesions were in general smaller and subcortically distributed (Figure 1). Two in this group had hemorrhagic strokes, whereas all others had ischemic strokes. A Mann-Whitney U test indicated that the lesion volume was significantly larger in the AOS group (Mdn = 121.9 cm3) than in the No SLI group (Mdn = 8.5 cm3), U = 2.0, *p* = 0.002 (Table 3). No significant correlation existed between lesion volume and any of the behavioral scores in the AOS group (at A1; ASRS rho = −0.35; A-ning rho = 0.18; BNT rho = 0.24; NVOA rho = −0.35). None of the participants in the No SLI group had a lesion overlap with any of the selected ROIs. In the AOS group, three (33%) of the participants had over 25% damage to the left vPMC, seven (78%) had over 25% damage to the left aINS, and seven (78%) to the left IFG. As shown in Table 4, several had over 25% damage to more than one ROI. There was no significant correlation between lesion volume and measures of FC in the selected network. A non-significant trend was found for the left aINS and left vPMC at the 6-months follow-up (rho = 0.65, *p* = 0.06).

**Table 3:** Demographic and clinical characteristics at the first assessment.

**Variables**			**AOS group**	**No SLI group**	**Group diff.^a^,^b^**
			**(*n* = 9)**	**(*n* = 6)**	
Age	Mean (SD)		49.6 (11.3)	56.7 (5.1)	0.237^a^
	Median		52	56.5	
Sex	*N (%)*	Females	2 (22)	1 (17)	0.792^b^
		Males	7 (78)	5 (83)	
Lesion volume cm3	Mean (SD)		133.0 (88.3)	14.4 (16.8)	0.002^a^*
	Median		121.9	8.5	
Stroke type	*N (%)*	Ischemic	8 (89)	4 (67)	0.292^b^
		Haemorrhagic	1 (11)	2 (33)	
FM-UE	Mean (SD)		11.1 (19.2)	44.8 (16.2)	0.013^a^*
	Median		2	51.5	

AOS group, participants with apraxia of speech, aphasia, and non-verbal oral apraxia; SLI, Speech-Language Impairment; FM-UE, Fugl-Meyer assessment for the upper extremity, max 60 p, cut-off level severe-moderate impairment 19 ± 2 points, moderate–mild impairment 47 ± 2 points.

a Mann-Whitney U test,

b Pearson Chi-Square, *p* < 0.05.

**Table 4:** Percentage lesion overlap with the selected ROIs in participants with AOS, aphasia, and NVOA.

**ID**	**Lesion overlap (%) to ROIs**			**Number of ROIs with >25% overlap**
	**vPMC**	**aINS**	**IFG**
1	5	31	30	2
2	0	100	100	2
3	0	10	0	0
4	16	100	100	2
5	0	28	0	1
6	94	96	100	3
7	0	0	44	1
8	85	33	100	3
9	100	100	100	3
*N (%)* with lesion overlap >25%	3 (33%)	7 (78%)	7 (78%)	

All regions refer to left hemisphere. IFG, inferior frontal gyrus; aINS, anterior insula; vPMC, ventral premotor cortex.

### Characterization of behavioral data and recovery in participants with AOS, aphasia and NVOA

At the early assessment (A1), six participants in the AOS group had very severe (global) aphasia, one a severe Broca's aphasia, and two a moderately severe Broca's aphasia. The mean A-ning score was 57.4 which corresponds to severe aphasia. The majority showed almost no naming ability; the median BNT score was 0, and the mean score 8.8. The ASRS total score ranged between 9 (indicating mild AOS) up to 44 (severe AOS). The mean ASRS score was 24.2 which indicates moderate to marked AOS (23). All had NVOA at this time point, several to a severe degree (Table 5).

**Table 5:** Clinical data and behavioral results for participants with AOS, aphasia, and NVOA.

**ID**	**Sex**	**Stroke**	**Age**	**Les. vol. cm3**	**ASRS**			**BNT**			**A-ning**			**NVOA**		
					**A1**	**A2**	**Rec.%**	**A1**	**A2**	**Rec.%**	**A1**	**A2**	**Rec.%**	**A1**	**A2**	**Rec.%**
1	F	I	52	30.6	44	35	20	0	21	35	35	95	32	16	17	6
2	M	I	65	121.9	18	15	17	1	2	2	36	46	5	6	7	4
3	M	I	57	32.9	23	15	35	0	5	8	26	86	31	2	9	23
4	F	I	39	117.1	22	14	36	12	38	54	70	120	33	17	20	20
5	M	H	31	167.7	17	9	47	49	51	18	134	202	79	18	30	86
6	M	I	39	175.6	28	18	36	0	44	73	38	173	74	5	21	59
7	M	I	61	160.2	9	2	78	17	43	60	150	190	57	12	20	40
8	M	I	54	73.1	32	23	28	0	49	82	18	133	57	26	30	67
9	M	I	48	317.8	25	24	4	0	0	0	10	25	7	0	0	0
Mean			49.6	133.0	24.2	17.2	33.4	8.8	28.1	36.9	57.4	118.9	41.7	11.3	17.1	33.9
(SD)			(11.3)	(88.3)	(9.9)	(9.4)	(21.0)	(16.3)	(21.2)	(31.5)	(50.9)	(62.1)	(26.7)	(8.6)	(10.1)	(30.8)
Median			52	121.9	23	15	35	0	38	35	36	120	33	12	20	23

F, female; M, male; I, ischemic stroke; H, hemorrhagic stroke; ASRS, Apraxia of Speech Rating Scale, max 52, cut-off value for AOS ≥ 8 points; BNT, Boston Naming Test, max 60; A-ning, Neurolinguistic Aphasia Examination, total score results and severity classification. Very severe/Global ≤ 42, Severe ≤ 82, Moderate ≤ 170, Moderate/mild ≤ 192, Mild ≤ 208, No Aphasia > 208, maximum 220 points. NVOA, Non-verbal oral apraxia protocol, max 32, cut-off value for NVOA diagnosis <29 points.

At six months follow-up (A2), total scores from all assessment instruments demonstrated statistically significant improvements (Wilcoxon signed-rank test ASRS Z = −2.67, *p* = 0.007; A-ning Z = −2.66, *p* = 0.008; BNT Z = −2.52, *p* = 0.012; NVOA Z = −2.52, *p* = 0.012). 1 participant recovered 78% and showed an almost complete AOS recovery with an ASRS score of 2, that is, below the recommended cut-off value at 8 points, while a majority of the remaining still had a moderate AOS. Two participants no longer had NVOA according to the result of the assessment.

The individual degrees of recovery covered a wide range in all behavioral assessments. The AOS recovery varied between 4 and 78%, with a mean value of 33%. The mean values for the other clinical recovery percentages were similar, with NVOA recovery of 34% and A-ning recovery of 42%. All speech and language results for each participant are presented in Table 5. The results from all seven domains in A-ning Neurolinguistic Aphasia Examination for each participant are provided in Supplementary Table S1.

### Functional connectivity comparison between groups

**Figure 2:**
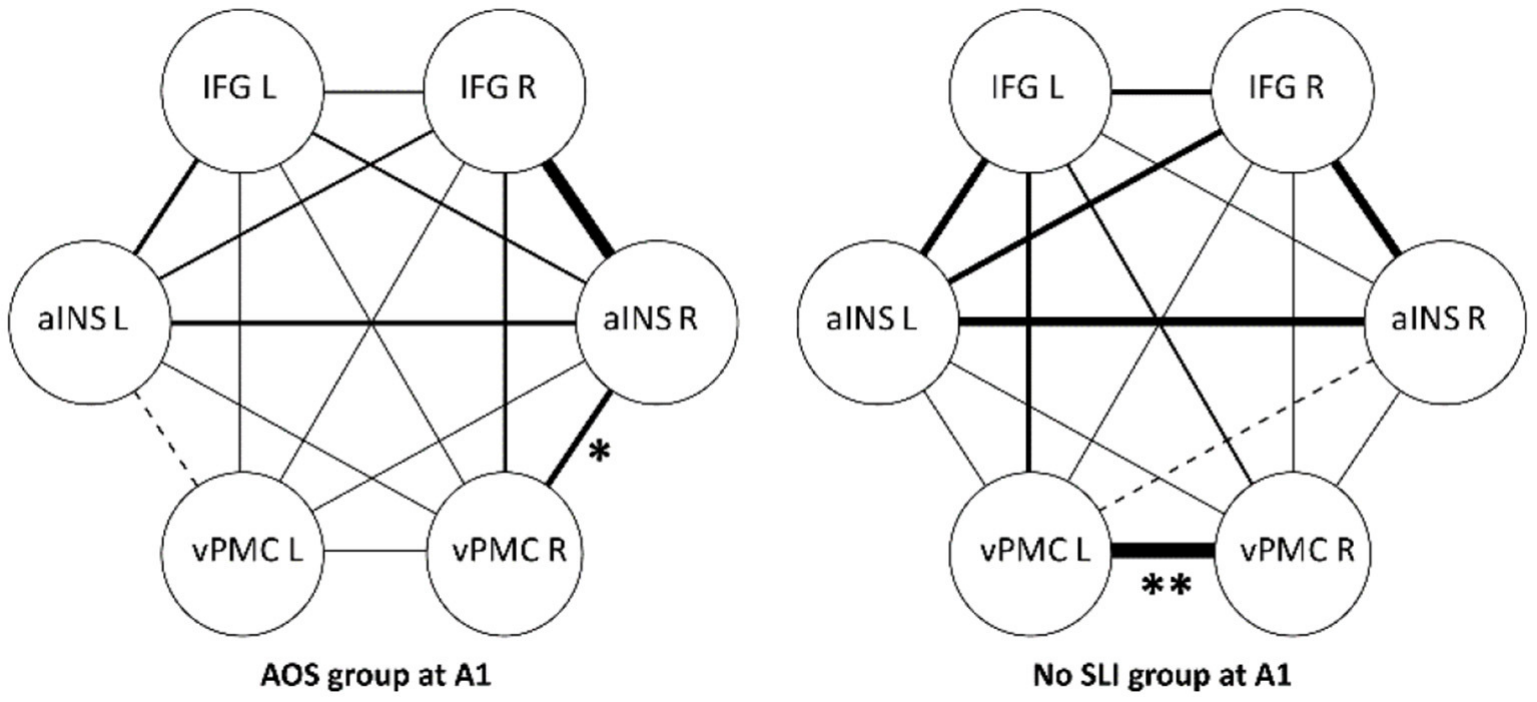
Functional connectivity strength between ROIs at A1, group comparison. IFG, inferior frontal gyrus; aINS, anterior insula; vPMC, ventral premotor cortex. Significant group differences marked with *(*p* < 0.05) and **(*p* < 0.01) in the figure. Line thickness illustrates degree of FC strength.

At A1, reduced FC was observed between all three interhemispheric homotopic ROIs in the AOS group compared to the No SLI group. The difference was significant for interhemispheric vPMC connectivity (Fisher's z-score 0.08 vs. 0.78, Mann-Whitney U test *p* = 0.001) while the reduced interhemispheric IFG connectivity in the AOS group showed a non-significant trend (Fisher's z-score 0.13 vs. 0.31, Mann-Whitney U test *p* = 0.08) and the interhemispheric aINS difference was the least prominent (Fisher's z-score 0.19 vs. 0.44, Mann-Whitney U test *p* = 0.16). In the non-lesioned hemisphere, participants in the AOS group displayed a significantly stronger intrahemispheric connectivity between the right aINS and right vPMC compared to the No SLI group (Fisher's z-score 0.32 vs. 0.06, Mann-Whitney U test *p* = 0.025). The strongest connectivity in the AOS group was found between the right IFG and right aINS (Fisher's z-score 0.57) (Table 6, Figure 2).

**Table 6:** Functional connectivity at A1, comparison between groups.

**Functional connectivity strength (Fisher's Z)**						
	**AOS group (*****n*** = **9)**		**No SLI group (*****n*** = **6)**			
	**Mean (SD)**	**Median**	**Mean (SD)**	**Median**	**^a^Group diff**.	**Effect size (*r)***
**Interhemispheric homotopic**						
IFG L–IFG R	0.130 (0.174)	0.132	0.314 (0.179)	0.332	0.077	0.46
aINS L–aINS R	0.187 (0.280)	0.170	0.435 (0.268)	0.372	0.157	0.36
vPMC L–vPMC R	0.079 (0.154)	0.039	0.777 (0.185)	0.756	0.001^**^	0.82
**Intrahemispheric Left**						
IFG L–aINS L	0.211 (0.200)	0.202	0.343 (0.233)	0.333	0.409	0.21
IFG L–vPMC L	0.109 (0.180)	0.183	0.189 (0.118)	0.164	0.637	0.12
aINS L–vPMC L	−0.007 (0.223)	0.025	0.067 (0.089)	0.099	0.479	0.18
**Intrahemispheric Right**						
IFG R–aINS R	0.571 (0.232)	0.541	0.416 (0.136)	0.413	0.238	0.30
IFG R–vPMC R	0.264 (0.212)	0.188	0.084 (0.142)	0.096	0.125	0.39
aINS R–vPMC R	0.321 (0.191)	0.354	0.061 (0.151)	0.053	0.026^*^	0.58
**Interhemispheric heterotopic**						
IFG L–aINS R	0.164 (0.279)	0.007	0.060 (0.182)	0.075	0.409	0.21
IFG L–vPMC R	0.023 (0.204)	0.046	0.134 (0.146)	0.179	0.288	0.27
IFG R–aINS L	0.150 (0.204)	0.144	0.309 (0.174)	0.306	0.125	0.40
IFG R–vPMC L	0.072 (0.152)	0.135	0.071 (0.148)	0.085	0.906	0.03
aINS L–vPMC R	0.050 (0.192)	0.060	0.001 (0.158)	0.036	0.814	0.06
aINS R–vPMC L	0.077 (0.194)	0.034	−0.059 (0.093)	−0.057	0.077	0.46

a Mann-Whitney U test,

* Significant at *p*-level < 0.05,

** Significant at *p*-level < 0.01. *r*, Mann-Whitney U test Effect Size.

At A2, the interhemispheric vPMC connectivity was still stronger in the No SLI group in comparison to the AOS group, but this did not reach significance (Fisher's z-score 0.46 vs. 0.12, Mann-Whitney U test *p* = 0.059). The earlier significantly stronger RH connection between aINS and vPMC in the AOS group was slightly reduced, while it had increased among participants with no SLI. A significant group difference was found between the right IFG and left aINS; this connection had decreased since A1 among AOS participants and increased in the No SLI group (Fisher's z-score 0.09 vs. 0.37, Mann-Whitney U test *p* = 0.034).

### Relation between FC and severity of AOS, aphasia and NVOA

At the first assessment, no significant correlations between FC and any behavioral score results were found. A non-significant trend for an association was present between interhemispheric IFG connectivity and A-ning, ASRS, and BNT results (rho = 0.79, *p* = 0.010; rho = −0.61, *p* = 0.081; rho = 0.63, *p* = 0.068 respectively), but none of these survived an FDR correction for multiple comparisons set at *q* < 0.003. The relation between interhemispheric vPMC connectivity and the ASRS total score was only moderate (rho −0.49, *p* = 0.177) (Table 7).

**Table 7:** Relation between FC and behavioral results at the first assessment (A1).

**FC at Assessment 1**	**ASRS A1**	**A-ning A1**	**BNT A1**	**NVOA A1**
IFG L–IFG R	−0.61	0.79	0.63	0.21
IFG L–aINS L	−0.59	0.59	0.39	−0.11
IFG L–aINS R	−0.29	0.49	0.32	0.13
IFG L–vPMC L	0.25	−0.31	−0.32	−0.21
IFG L–vPMC R	−0.46	0.49	0.39	−0.11
IFG R –aINS L	−0.06	−0.13	−0.12	0.07
IFG R –aINS R	0.14	−0.43	−0.10	0.33
IFG R–vPMC L	−0.19	0.14	0.01	−0.43
IFG R–vPMC R	0.21	−0.23	−0.23	0.26
aINS L–aINS R	−0.59	0.31	0.43	0.24
aINS L–vPMC L	−0.08	−0.14	−0.01	0.13
aINS L–vPMC R	−0.21	0.09	0.01	−0.06
aINS R–vPMC L	−0.11	0.36	0.05	−0.08
aINS R–vPMC R	−0.23	0.01	0.12	0.26
vPMC L–vPMC R	−0.49	0.34	0.39	−0.14

Spearman's rho, FDR-corrected significance level *q* < 0.003.

At six months follow-up (A2), a significant correlation between ASRS result and interhemispheric aINS connectivity was found (rho = −0.88, *p* = 0.002, *q* < 0.003). A strong but non-significant correlation was found between ASRS total score and FC strength between left IFG and right aINS (rho = −0.80, *p* = 0.009) and with interhemispheric homotopic vPMC FC (rho = −0.79*, p* = 0.012). There were no significant correlations between FC strength and A-ning (covering all language domains), BNT (naming ability), or NVOA (Table 8).

**Table 8:** Relation between FC and behavioral results at the second assessment (A2).

**FC at Assessment 2**	**ASRS A2**	**A-ning A2**	**BNT A2**	**NVOA A2**
IFG L–IFG R	−0.29	0.22	−0.02	−0.02
IFG L–aINS L	−0.40	−0.27	−0.47	−0.50
IFG L–aINS R	−0.80	0.67	0.43	0.39
IFG L–vPMC L	0.07	−0.18	−0.38	−0.44
IFG L–vPMC R	0.06	0.05	−0.02	−0.08
IFG R –aINS L	−0.23	−0.22	−0.45	−0.46
IFG R –aINS R	0.01	0.13	0.17	0.20
IFG R–vPMC L	−0.13	0.47	0.30	0.23
IFG R–vPMC R	0.23	0.12	0.32	0.34
aINS L–aINS R	−0.88*	0.43	0.27	0.22
aINS L–vPMC L	−0.49	0.43	0.20	0.10
aINS L–vPMC R	0.25	−0.05	0.15	0.14
aINS R–vPMC L	−0.26	0.53	0.40	0.33
aINS R–vPMC R	−0.10	0.47	0.57	0.55
vPMC L–vPMC R	−0.79	0.76	0.67	0.64

Spearman's rho, *significant at FDR level *q* < 0.003.

### Longitudinal changes in FC in patients with AOS

**Figure 3:**
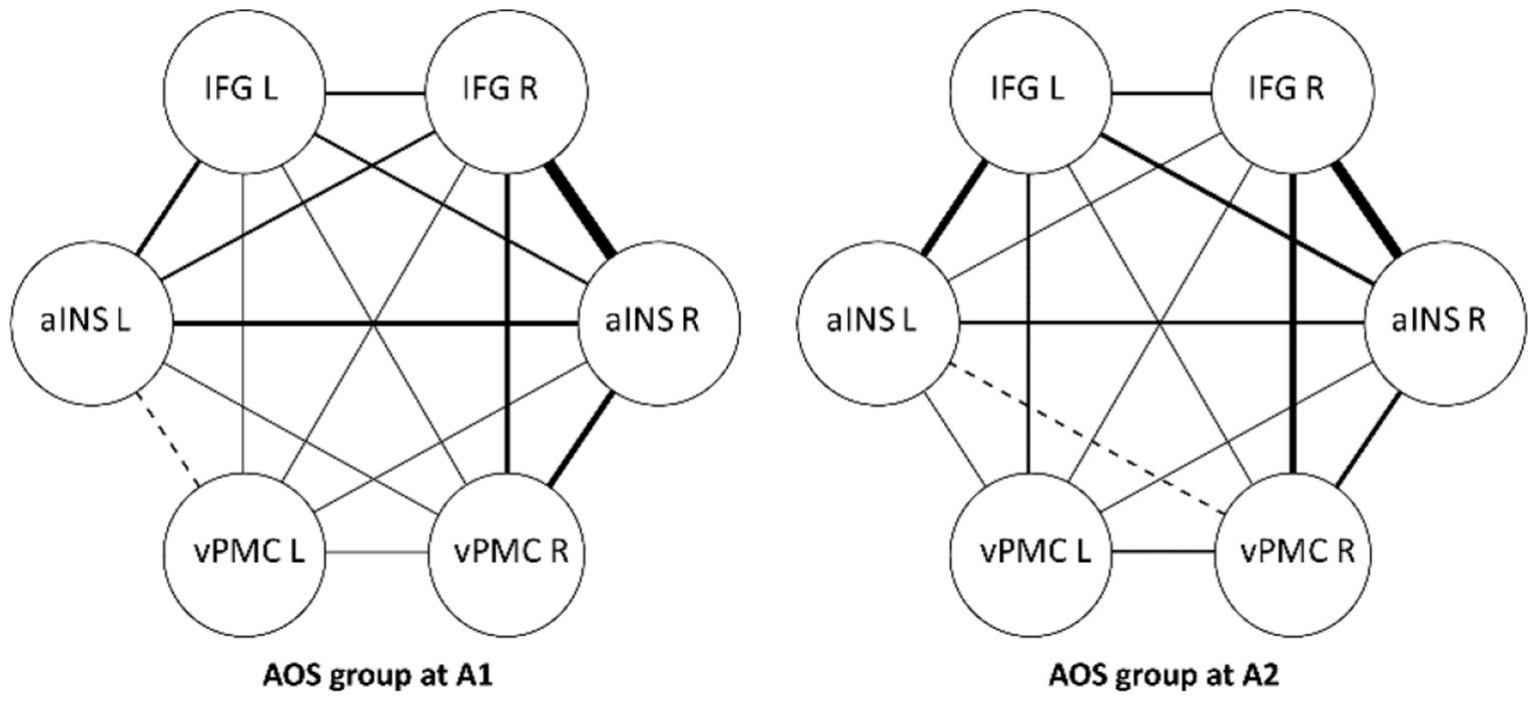
Functional connectivity strength between ROIs, within group differences. Participants with AOS, aphasia and NVOA. IFG, inferior frontal gyrus; aINS, anterior insula; vPMC, ventral premotor cortex. Line thickness illustrates degree of FC strength.

At a group level in the AOS group, no significant changes in FC were found between the two assessment occasions. All three interhemispheric homotopic seed connections remained low. Qualitatively, the largest increase was found between the left IFG and left aINS (Fisher's z-score 0.21 vs. 0.39, *p* = 0.11). Although slightly reduced at A2, the strongest connection was still found between right IFG and right aINS (Fisher's z-score 0.50) (Table 9, Figure 3).

**Table 9:** Functional connectivity at first and second assessment, participants with AOS (*n* = 9).

		**Functional connectivity strength (Fisher's Z)**			
	**Assessment 1**		**Assessment 2**			
	**Mean (SD)**	**Median**	**Mean (SD)**	**Median**	**^a^Within group diff**.	**Effect size (*r)***
**Interhemispheric homotopic**						
IFG L–IFG R	0.130 (0.174)	0.132	0.134 (0.160)	0.127	0.594	0.14
aINS L–aINS R	0.187 (0.280)	0.170	0.178 (0.316)	0.094	0.859	0.05
vPMC L–vPMC R	0.079 (0.154)	0.039	0.123 (0.257)	0.178	0.515	0.17
**Intrahemispheric LEFT**						
IFG L–aINS L	0.211 (0.200)	0.202	0.394 (0.174)	0.376	0.110	0.41
IFG L–vPMC L	0.109 (0.180)	0.183	0.179 (0.154)	0.159	0.515	0.17
aINS L–vPMC L	−0.007 (0.223)	0.025	0.024 (0.164)	0.105	0.767	0.08
**Intrahemispheric RIGHT**						
IFG R–aINS R	0.571 (0.232)	0.541	0.496 (0.205)	0.498	0.260	0.29
IFG R–vPMC R	0.264 (0.212)	0.188	0.296 (0.167)	0.331	0.515	0.16
aINS R–vPMC R	0.321 (0.191)	0.354	0.216 (0.254)	0.237	0.214	0.32
**Interhemispheric heterotopic**						
IFG L–aINS R	0.164 (0.279)	0.007	0.213 (0.230)	0.281	0.859	0.05
IFG L–vPMC R	0.023 (0.204)	0.046	0.097 (0.141)	0.082	0.314	0.26
IFG R–aINS L	0.150 (0.204)	0.144	0.096 (0.226)	0.029	0.594	0.14
IFG R–vPMC L	0.072 (0.152)	0.135	0.044 (0.125)	0.071	0.953	0.02
aINS L–vPMC R	0.050 (0.192)	0.060	−0.037 (0.219)	0.000	0.441	0.20
aINS R–vPMC L	0.077 (0.194)	0.034	0.054 (0.211)	0.116	0.374	0.23

a Wilcoxon Signed Rank test, *p* = 0.05. *r* = Wilcoxon Signed Rank test Effect Size.

### Relation between behavioral recovery and FC

**Figure 4:**
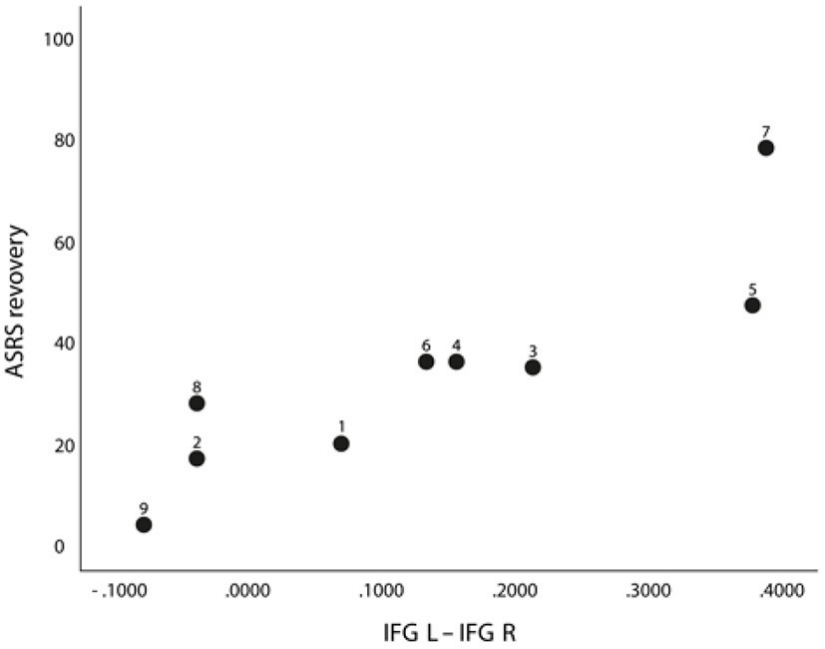
Correlation between ASRS recovery and FC between left and right IFG at A1 (rho = 0.92*, p* = 0.001/*q* = <0.003) in nine participants with AOS (with participant ID shown).

The interhemispheric homotopic IFG connectivity at the early assessment correlated strongly and positively with AOS recovery (rho = 0.92, *p* = 0.001). Thus, participants with higher FC here at 4 weeks after stroke showed more favorable AOS recovery to 6 months (Figure 4). This result remained significant when adding number of unaffected gray matter voxels in the IFG ROI as a covariate in the correlation analyses (partial *r* = 0.91, *p* = 0.002). The intrahemispheric connection between left aINS and left IFG was approaching a significant correlation with AOS recovery (rho = 0.81, *p* = 0.007), but did not reach the FDR corrected significance level set at *q* < 0.003. A-ning, BNT, and NVOA recovery did not correlate with any early FC result (Table 10).

**Table 10:** Correlation between recovery in behavioral measurements and functional connectivity between ROIs, results presented in descending order (based on the ASRS result).

**FC at Assessment 1**	**ASRS recovery**	**A-ning recovery**	**BNT recovery**	**NVOA recovery**
IFG L–IFG R	0.92^*^	0.56	0.26	0.55
IFG L–aINS L	0.81	0.45	0.29	0.54
IFG L–vPMC R	0.66	0.25	0.02	0.17
IFG L–aINS R	0.55	0.45	0.16	0.47
vPMC L–vPMC R	0.53	0.18	−0.26	0.18
aINS R–vPMC L	0.43	0.01	0.39	−0.05
aINS L–aINS R	0.36	0.08	0.23	0.35
IFG R–vPMC L	0.21	−0.18	0.04	−0.35
aINS L–vPMC R	0.20	−0.28	0.18	−0.08
aINS R–vPMC R	0.02	−0.15	0.24	0.13
IFG R–aINS L	−0.04	−0.10	0.24	0.22
IFG R–vPMC R	−0.21	−0.21	0.21	0.10
aINS L–vPMC L	−0.24	−0.32	0.21	−0.22
IFG L–vPMC L	−0.40	−0.38	−0.11	−0.42
IFG R–aINS R	−0.46	−0.25	−0.26	0.07

Spearman's rho, *significant at FDR level *q* < 0.003.

## Discussion

This study provides one of the first data sets presenting FC changes in a bilateral speech production network in patients with AOS in an early phase after stroke. The main finding was a strong correlation between the interhemispheric IFG connectivity and the individual AOS recovery. Participants with low FC between these seeds at 4 weeks post-stroke showed less recovery at 5 months than participants with higher FC (Figure 4). In line with findings by New et al. (36), participants with AOS in the present study also had a significantly reduced interhemispheric vPMC seed connectivity in comparison with LH lesioned participants without a speech-language impairment. The interhemispheric vPMC connectivity strength was however only moderately related to AOS severity at the early assessment, but nearly reached an FDR-corrected significant correlation at 6 months follow-up (rho = −0.79, *p* = 0.012). The strongest correlation between AOS severity and FC strength was found between interhemispheric aINS connectivity and the ASRS score result at 6 months (rho = −0.88, *p* = 0.002).

### Intra–and interhemispheric IFG functional connectivity and AOS recovery

The importance of the IFG in our results is in line with Hillis et al. (11), who reported that in patients in an acute stage after stroke, the presence of AOS was associated with hypoperfusion and/or infarct affecting Broca's area in the left hemisphere. Our result is also in keeping with meta-analyses by Turkeltaub et al. (59) who found a pattern of over activation both in left perilesional regions and in RH regions homotopic to the LH hemisphere speech-language network. Specifically, a stronger activation in the right IFG tended to be more often present in individuals with lesions in the left IFG than in those without a lesion in that area. Several studies investigating aphasia after stroke have also proposed that RH areas can contribute to the recovery (60, 61) and that the right IFG can support speech production when the homologous left side is lesioned (62, 63). In a longitudinal study of individuals with primary progressive aphasia and progressive AOS, worsening of AOS was significantly correlated with greater progressive cortical thinning in the right IFG (29). Our finding that interhemispheric IFG connectivity in the early phase after stroke may be an important predictor of AOS recovery fits with the Eickhoff model emphasizing the role of IFG as a starting point in speech production (19). Thus, even if areas in the vPMC are considered as the key region on which speech planning and programming depends, the present results underscore the central role of the left IFG and its intra- and interhemispheric connections to the rest of the speech-language network in determining recovery from AOS.

The reorganization of the speech-language network is however known to be more complex than just a direct reengagement of homologous regions (26). Skipper-Kallal et al. (64) proposed that the increased activity of the RH may stem from a cascading effect from one right hemisphere node taking over synapses from its left homotopic hemisphere counterpart. Nodes in the entire right network that are connected to this one node may then be activated and involved in compensation, resulting in broad patterns of increased activity. In accordance with this suggestion, participants with AOS in our study displayed a significantly stronger right sided intrahemispheric connectivity between the right aINS and right vPMC in comparison to LH lesioned without a speech-language impairment (Figure 2). New et al. (36) observed the same pattern in a comparison with healthy controls and suggested that reallocating functions from the lesioned hemisphere to homologous areas in the RH could be used in compensation for the interrupted LH networks. They also speculated that for some patients with AOS, an upregulation of the right vPMC region may not be sufficient. To compensate, an increased activation of the right aINS may also occur (36). Ramage et al. (65) used the same data set as New et al. to study a network of brain regions considered as key regions for semantic and phonological processing in post stroke aphasia. Their results indicated that ipsilateral connections between temporal and frontal regions in both the left and the right hemisphere could predict access to semantic and phonological representations that serve as a basis for speech production. The findings from our study are in line with these interpretations and suggest that functional connectivity changes in both intra- and interhemispheric IFG connections can be involved in early AOS recovery.

### Functional connectivity in relation to severity of AOS, aphasia and NVOA

Despite the strong correlation between recovery of AOS and early interhemispheric IFG connectivity, the IFG connectivity strength did not correlate significantly with AOS severity early or late post-stroke. Unlike in the study by New et al. (36) that investigated mainly chronic AOS patients, we found that AOS severity also was not strongly associated to the bilateral vPMC connectivity strength at the early stage (rho = −0.49, *p* = 0.177). At the 6 months follow-up, however, a comparable pattern of strengthened connection between bilateral vPMC connectivity and AOS severity was found that almost reached an FDR corrected significance level (rho = −0.79, *p* = 0.012). At this time point, a significant correlation between bilateral aINS connectivity strength and AOS severity was also found (rho = −0.88, *p* = 0.002). In addition, a strengthened association was noticed between ASRS total score and FC strength between left IFG and right aINS (rho = −0.80, *p* = 0.009). These results accord with the current view of the importance of premotor regions for speech motor programming and the severity level of AOS, and further suggest that also the bilateral aINS connectivity may have an impact on AOS impairment level.

We found no significant correlations regarding FC between any of the selected ROIs in relation to behavioral results in the language domain or for NVOA. Given the recognized association between areas in IFG and expressive language, it could have been expected that the aphasia recovery would be closely connected to intra- and/or interhemispheric IFG connectivity strength and to the increased activation of the right IFG. However, the FC strength between the selected ROIs in our study displayed a closer connection to the measurements of AOS than those from the broad aphasia test (A-ning) and the naming test (BNT) (Table 10). As one possible explanatory factor, our selected ROI in the IFG area was in the posterior part, corresponding to the pars opercularis (Brodmann area (BA) 44) in Broca's area. This area has been proposed to play an important role in motor speech programming (66) whereas the pars triangularis, BA 45, in the anterior part has been suggested to play a greater role at a linguistic level of speech production (15, 67). As another important factor, our results were based on a small study sample (further adressed in the limitation section) and needs to be confirmed in larger samples.

### Comparison of FC between LH stroke patients with AOS and LH lesioned patients without speech-language impairment

As a comparison group in this study, a small sample of LH stroke patient without a speech-language impairment recruited from the ProHand study cohort was used. This option allowed us to further investigate if the FC pattern observed by New et al. (36) in healthy subjects also is visible in early LH stroke patients without AOS and aphasia. The comparison group had significantly smaller and mainly subcortical lesions, not affecting any of the selected cortical ROIs. In accordance with the previous findings, the AOS participants in our study had a significantly reduced interhemispheric homotopic vPMC seed connection in comparison with LH lesioned without a speech-language impairment. This reduced connectivity stayed over time and was only slightly strengthened at the 6 months follow-up. Considering that premotor areas, both in BA6 (corresponding to our ROI in vPMC) and in the posterior part of the IFG (corresponding to our IFG ROI) have been suggested to be involved in the hand motor movement (68, 69) signs of a disturbed network connectivity between the selected ROIs might have been present also in the No SLI group. However, in our No SLI group, we found a FC pattern similar to that observed by New et al. (36) in healthy individuals.

## Methodological considerations and study limitations

The application of rs-fMRI in stroke research has increased during the past decade. Stroke lesions do however present some challenges for the FC analyses. There is no total consensus on how the BOLD signal should be preprocessed and how to analyze data from stroke patients with large lesions affecting selected ROIs (70, 71). For transparency, we therefore chose to report all lesion overlap to our selected ROIs. As can be seen in Table 4, most participants with AOS in the present study had over 25% lesion overlap to any of the selected ROIs, while the comparison group without speech-language impairment mainly had isolated subcortical lesions with no engagement of the cortical nodes at study. However, the vPMC was the region with the lowest percentage lesion overlap among our selected ROIs. The significantly reduced FC between bilateral vPMC can therefore not be interpreted as merely a direct consequence of a lesion overlap. As also can be seen in Table 4, seven out of the nine included in the AOS group had over 25% damage to the left IFG, and two of them (ID 6 and 9 in Figure 4) had an almost total overlap to all three selected ROIs. However, Figure 4 shows that the relation between AOS recovery and interhemispheric IFG FC was maintained even in the patients with more than 25% of lesion-overlap to the left IFG (i.e., the relation was visible even in patients with the lowest FC values). To be added, we cannot rule out that other cortical and subcortical lesion profiles could affect the connectivity in speech and networks and the selected ROIs. The limited number of subjects included in this study prevented further testing of lesion-behavior relationships, as for example by voxel-based lesion symptom mapping analysis (VLSM).

Recovery in this study was primarily investigated focusing on the amount of change, defined as the percentage that a participant improves over time on a test in relation to the possible maximum improvement on that specific measure. This method has been applied in several studies as an alternative to investigate and describe the inter-individual variability in stroke recovery, as for example by Lazar et al. (72) and Marchi et al. (73). However, it has been recognized that the sensitivity to change may differ among different behavioral assessment instruments. To further investigate accuracy and predictive value of the recovery analysis and the role of bilateral IFG in AOS recovery, we therefore compared the behavioral test results at A2 with the initial FC at A1. Also in this analysis, the same pattern emerged with the bilateral IFG at the subacute phase being the strongest predictor of the ASRS result at A2 (rho = −0.803, *p* = 0.009). All these results are presented in the Supplementary Table S2.

Since all participants with AOS in this study also had aphasia and NVOA at the early assessment, it was not possible to make a direct group comparison to patients with isolated aphasia. In clinical settings, this co-occurrence is very common. According to Duffy (3), patients with Broca's or non-fluent aphasia often also have AOS. We therefore chose to compare and report how clinical results in the different domains, both severity/score results and recovery of AOS, aphasia and NVOA, related to the FC results.

Considering earlier observed limitations in assessing individuals with severe AOS with the ASRS (48), the choice to use it in this study may seem irrational. However, because of the increasing support for the ASRS, becoming a standard instrument in the AOS literature (45), the ASRS was still our choice. To adjust the ratings in line with the severity level in the study sample, a number of modifications presented in Table 1 were applied. High attention was paid to the influence of comorbidity and risk for perceptual overlap for every item rating, and all ratings with the ASRS were carried out in consensus by at least two raters. An additional point concerns the use of the ASRS 2.0, and not the current version ASRS 3.0. The ASRS 2.0 consists of the same 13 items as the ASRS 3.0 and uses the same 5-point grading scale with operationalized descriptors for each rating level. The only difference between the two versions is that ASRS 2.0 is organized in three different sections according to whether the features are considered as (a) primary distinguishing features (rare overlap with dysarthria or aphasia), (b) distinguishing features unless dysarthria present and (c) distinguishing features unless aphasia and/or dysarthria are present, while the items in ASRS 3.0 are organized in three sections according to whether they are (a) articulatory features, (b) prosodic features or (c) other features. In the present study, the ASRS 2.0 total score was used as an index of AOS severity. We thereby believe that the same result would have been found by use of the ASRS 3.0 and that the obtained result is a valid measure of the severity of AOS in this study cohort.

Several limitations to this study need to be acknowledged. This report is based on the longitudinal follow-up of nine stroke patients. This is a rather small participant sample which inevitably limits the statistical strength in the performed analyses. Our results should thus be replicated in larger samples. However, the use of the same ROIs as New et al. (36) made it possible for us to add new data to previously published results and allowed a direct comparison with their findings. Hopefully, the results and tendencies observed in this small study sample may also be of value for future studies. Secondly, the cohort in this study was relatively young compared to the overall stroke population. There was also an imbalance in gender, with more men than women included. Thirdly, all participants with AOS received speech-language therapy with four to five sessions a week during inpatient care and two to three times a week after discharge provided by the regional rehabilitation team. The exact dose, frequency and focus of the therapy sessions were however not possible to control for. In addition, the therapeutic effects and brain plasticity mechanisms in relation to improved speech-language functions and changes in FC cannot be disentangled. Finally, we only included three bilateral cortical seed regions in our analyses out of a much larger network related to speech production. A whole-brain analysis of FC might have revealed other key functional connectivity patterns important for AOS recovery. Investigation of subcortical areas and disruption of structural connectivity in white matter bundles should also be of highest interest. Future studies should consider adding other regions to the analyses that are regarded as key nodes in the speech production network.

## Conclusions

To our knowledge, this study provides the first longitudinal FC data in AOS, spanning from the early to the chronic phase post stroke. Despite the limited sample size, the results clearly indicated that early interhemispheric IFG connectivity may be a strong predictor of AOS recovery, thus adding to our knowledge about the neural mechanisms underlying AOS and to the recovery process. The results confirmed earlier findings regarding the role of the ventral premotor region in speech motor programming and severity level of AOS, and suggest that the involvement of the bilateral inferior frontal gyrus and an increased activation of homologous areas in the right hemisphere speech production network may contribute to the recovery of AOS.

## Data availability statement

The raw data supporting the conclusions of this article will be made available by the authors, without undue reservation.

## Ethics statement

The studies involving human participants were reviewed and approved by the Regional Ethical Review Board in Stockholm. The patients/participants provided their written informed consent to participate in this study.

## Author contributions

Study concept and design: PL, PÖ, ES, JP, and HH. Study supervision: PL, PÖ, and ES. Data collection: HH and JP. Analysis and interpretation of data: HH, PL, PÖ, JP, ES, CD, and JB. Manuscript draft and manuscript writing: HH. Critical revision of manuscript: PL, PÖ, ES, JP, CD, and JB. All authors have read and approved the submitted version of the manuscript.

## Funding

This work was supported by the Promobilia Foundation and by Karolinska Institute and Karolinska University Hospital.

## Conflict of interest

The authors declare that the research was conducted in the absence of any commercial or financial relationships that could be construed as a potential conflict of interest.

## Publisher's note

All claims expressed in this article are solely those of the authors and do not necessarily represent those of their affiliated organizations, or those of the publisher, the editors and the reviewers. Any product that may be evaluated in this article, or claim that may be made by its manufacturer, is not guaranteed or endorsed by the publisher.
