# Supplementary material for: Longitudinal changes in functional connectivity in speech motor networks in apraxia of speech after stroke
Source: Front Neurol. 2022 Nov 30;13:1013652. doi: 10.3389/fneur.2022.1013652 (PMC9748434; doi:10.3389/fneur.2022.1013652)
Supplement: Supplementary file 1 [file Data_Sheet_1.docx]

**SUPPLEMENTARY MATERIAL**

**Table A**. A-ning results at four weeks after stroke (A1) and at 6-months follow-up (A2)

|  | **Total**  **score Index** | **A.**  **Oral expr.** | **B. Repetition** | **C.**  **Auditory comp.** | **D.**  **Reading comp.** | **E.**  **Reading aloud** | **F.**  **Dictation** | **G. Informative writing** |
| --- | --- | --- | --- | --- | --- | --- | --- | --- |
| ID | **A1/A2** | **A1/A2** | **A1/A2** | **A1/A2** | **A1/A2** | **A1/A2** | **A1/A2** | **A1/A2** |
| 1 | 0.8/2.2 | 0.6/2.3 | 0.8/2.0 | 0.9/2.4 | 0.9/3.1 | 1.0/2.5 | 1.3/ | 0.3/ |
| 2 | 0.8/1.0 | 1.3/1.5 | 2.0/1.9 | 1.0/1.0 | 0.1/0.5 | 0.0/0.3 | 0 | 0.3 |
| 3 | 0.6/2.0 | 0.3/2.3 | 1.5/3.3 | 0.5/1.1 | 0.3/2.3 | 1.3/2.5 | 0.3 | 0 |
| 4 | 1.6/2.7 | 1.8/3.0 | 2.0/3.0 | 1.8/3.6 | 2.4/2.6 | 0.3/2.3 | 1 | 0.5 |
| 5 | 3.0/4.6 | 2.9/4.4 | 3.9/4.3 | 2.8/4.9 | 2.8/4.9 | 4.0/4.3 | 2.8 | 2.3 |
| 6 | 0.9/3.9 | 0.1/3.8 | 0.4/3.8 | 2.3/4.3 | 1.4/4.6 | 0.0/3.8 | 1.3 | 0 |
| 7 | 3.4/4.3 | 3.1/4.4 | 4.1/4.8 | 3.8/4.1 | 3.8/4.8 | 4.3/4.3 | 2.8 | 1 |
| 8 | 0.4/3.0 | 0.1/3.1 | 0.5/3.6 | 0.9/2.9 | 0.1/3.3 | 0.8/3.0 | 0 | 0.5 |
| 9 | 0.2/0.6 | 0.1/0.1 | 0.3/0.7 | 0.5/0.9 | 0.4/1.6 | 0.0/0.5 | 0 | 0 |

A-NING = A-ning Neurolinguistic Aphasia Examination, severity index classification, range 0 -5: Very severe/Global ≤ 0.9, Severe ≤ 1.8, Moderate ≤ 3,8, Moderate/mild ≤ 4.4, Mild ≤ 4.7), No Aphasia > 4.8.

**Table B.** Relation between functional connectivity (FC) at 4 weeks after stroke (A1) and clinical result at 6 months follow-up (A2)

| **FC at Assessment 1** | **ASRS A2** | **A-ning A2** | **BNT A2** | **NVOA A2** |
| --- | --- | --- | --- | --- |
| IFG L− IFG R | **-0.80** | **0.70** | **0.48** | **-0.42** |
|  | 0.009 | 0.035 | 0.185 | 0.263 |
| IFG L− aINS L | **-0.77** | **0.55** | **0.37** | **-0.31** |
|  | 0.015 | 0.123 | 0.330 | 0.407 |
| IFG L− aINS R | **-0.35** | **0.59** | **0.40** | **0.30** |
|  | 0.358 | 0.097 | 0.284 | 0.434 |
| IFG L−vPMC L | **0.44** | **-0.30** | **-0.38** | **-0.42** |
|  | 0.235 | 0.431 | 0.306 | 0.263 |
| IFG L−vPMC R | **-0.62** | **0.28** | **0.10** | **0.08** |
|  | 0.076 | 0.458 | 0.797 | 0.838 |
| IFG R −aINS L | **-0.16** | **-0.07** | **0.08** | **0.14** |
|  | 0.674 | 0.864 | 0.831 | 0.721 |
| IFG R− aINS R | **0.21** | **-0.25** | **0.00** | **0.03** |
|  | 0.580 | 0.515 | 1.000 | 0.940 |
| IFG R – vPMC L | **-0.20** | **-0.13** | **-0.35** | **-0.35** |
|  | 0.595 | 0.731 | 0.354 | 0.356 |
| IFG R – vPMC R | **0.07** | **-0.18** | **0.03** | **0.11** |
|  | 0.855 | 0.635 | 0.932 | 0.770 |
| aINS L− aINS R | **-0.63** | **0.28** | **0.23** | **0.21** |
|  | 0.066 | 0.458 | 0.544 | 0.578 |
| aINS L – vPMC L | **-0.05** | **-0.25** | **-0.17** | **-0.10** |
|  | 0.906 | 0.515 | 0.667 | 0.804 |
| aINS L – vPMC R | **-0.34** | **-0.12** | **-0.20** | **-0.17** |
|  | 0.370 | 0.764 | 0.604 | 0.656 |
| aINS R – vPMC L | **-0.32** | **0.10** | **-0.05** | **-0.02** |
|  | 0.396 | 0.797 | 0.898 | 0.957 |
| aINS R – vPMC R | **-0.32** | **-0.05** | **0.07** | **0.13** |
|  | 0.396 | 0.898 | 0.864 | 0.737 |
| vPMC L – vPMC R_ | **-0.54** | **0.23** | **0.05** | **-0.01** |
|  | 0.132 | 0.544 | 0.898 | 0.974 |

*Note*: Spearman’s rho, FDR-corrected significance level *q* < 0.003. ASRS = Apraxia of Speech Rating Scale, A-ning = Neurolinguistic aphasia Examination, BNT = Boston Naming Test, NVOA = Nonverbal oral apraxia protocol.
